# Supplementary material for: Cannabis companies and the sponsorship of scientific research: A cross-sectional Canadian case study
Source: PLoS One. 2023 Jan 10;18(1):e0280110. doi: 10.1371/journal.pone.0280110 (PMC9831296; doi:10.1371/journal.pone.0280110)
Supplement: S1 Table — (DOCX) [file pone.0280110.s002.docx]

**S2 Table. Sampling and search strategies**

**List of sampled parent companies and their subsidiaries**

| **Parent name** | **Parent Aliases** | **Subsidiaries** | **Subsidiary Aliases** |
| --- | --- | --- | --- |
| AgMedica Bioscience | AgriMed Botanicals | |  |
| Aleafia | Wyn Metals |  |  |
| Aleafia |  | Canabo Medical |  |
| Aleafia |  | Emblem |  |
| Aleafia |  | GrowWise Health |  |
| Aleafia |  | Flying High Brands | |
| Aqualitas |  |  |  |
| Aqualitas | Sindica Global Institute for Cannabis Research & Innovation | | |
| Aqualitas | Reef Organic |  |  |
| Atlas Biotechnologies | |  |  |
| Atlas Biotechnologies | | Atlas Growers |  |
| Atlas Biotechnologies | | Atlas Thrive |  |
| Aurora Cannabis | Milk Capital |  |  |
| Aurora Cannabis |  | Aurora Nordic Cannabis | |
| Aurora Cannabis |  | H2 Biopharma |  |
| Aurora Cannabis |  | Peloton Pharmaceuticals | |
| Aurora Cannabis |  | Reliva |  |
| Aurora Cannabis |  | Whistler Medical Marijuana | |
| Aurora Cannabis |  | ACB Captive Insurance | |
| BeeHigh Vital Elements | BeeHighVE |  |  |
| BeeHigh Vital Elements | | Wild Cove Smoke |  |
| BeeHigh Vital Elements | | BH! |  |
| Benchmark Botanics | |  |  |
| Benchmark Botanics | | Potanicals Green Growers | |
| Benchmark Botanics | | Canada Bond Biotechnology | |
| Canada House Wellness Group | Abba Medix |  |  |
| Canada House Wellness Group | | Canada House Clinics | |
| Canada House Wellness Group | | Marijuana For Trauma | |
| Canada House Wellness Group | | Knalysis Technologies | |
| Canada House Wellness Group | | The Longevity Project | |
| Canada House Wellness Group | | IsoCanMed |  |
| CannTrust |  |  |  |
| CannTrust |  | Elmcliffe Investments | |
| CannTrust |  | CTI Holdings |  |
| CannTX Life Sciences | |  |  |
| CannTX Life Sciences | | Steadystem Solutions | |
| CannTX Life Sciences | | Bower Therapies |  |
| CannTX Life Sciences | | Royal City Cannabis | |
| CannTX Life Sciences | | Solidus Standard |  |
| Canopy Growth | LW Capital Pool |  |  |
| Canopy Growth |  | Algarithm Ingredients | |
| Canopy Growth |  | Apollo Applied Research | |
| Canopy Growth |  | BATAVIA BIO PROCESSING | |
| Canopy Growth |  | C3 Cannabinoid Compound Company | |
| Canopy Growth |  | Beckley Canopy Therapeutics | |
| Canopy Growth |  | Canamo y Fibras Naturales | |
| Canopy Growth |  | BioSteel Sports Nutrition | |
| Canopy Growth |  | Coldstream Manufacturing | |
| Canopy Growth |  | Coldstream Real Estate Holdings | |
| Canopy Growth |  | East Coast Tween |  |
| Canopy Growth |  | EB TRANSACTION |  |
| Canopy Growth |  | EB Transaction |  |
| Canopy Growth |  | HIP Developments | |
| Canopy Growth |  | HIP NY Developments | |
| Canopy Growth |  | JuJu Joints |  |
| Canopy Growth |  | Lakessence |  |
| Canopy Growth |  | Les Serres Vert Cannabis | |
| Canopy Growth |  | POS |  |
| Canopy Growth |  | Spectrum Biomedical | |
| Canopy Growth |  | Spectrum Labs |  |
| Canopy Growth |  | Spectrum Therapeutics | |
| Canopy Growth |  | Storz & Bickel | Storz and Bickel |
| Canopy Growth |  | Tweed |  |
| Canopy Growth |  | THC Pharm GmbH Health Concept | |
| Canopy Growth |  | TWP |  |
| Canopy Growth |  | Wachstum Produce GP | |
| Canopy Growth |  | The Supreme Cannabis Company | Supreme Cannabis |
| Canopy Growth |  | AV Cannabis | Ace Valley |
| Cronos |  |  |  |
| Cronos |  | Hortican[Conflict of Interest Statements] | |
| Cronos |  | Peace Naturals Project | |
| Cronos |  | Original BC |  |
| Cronos |  | Zeus Cannabinoids | |
| Cronos |  | Redwood |  |
| Cronos |  | Thanos Holdings |  |
| Emerald Health | Thunderbird Biomedical; T-Bird Pharma | | |
| Emerald Health |  | Avalite Sciences |  |
| Emerald Health |  | Verdélite | Verdelite |
| Eve & Co | Eve and Co |  |  |
| Eve & Co |  | Natural MedCo |  |
| GOOD BUDS |  |  |  |
| GTEC Holdings | Black Birch Capital Acquisition; Capital Pool Company | | |
| GTEC Holdings |  | Alberta Craft Cannabis | |
| GTEC Holdings |  | GreenTec |  |
| GTEC Holdings |  | Grey Bruce Farms |  |
| GTEC Holdings |  | Spectre Labs |  |
| GTEC Holdings |  | Tumbleweed Farms | |
| GTEC Holdings |  | Zenalytic Laboratories | |
| Harvest One Cannabis | |  |  |
| Harvest One Cannabis | | United Greeneries |  |
| Harvest One Cannabis | | Satipharm |  |
| Harvest One Cannabis | | Dream Products |  |
| Harvest One Cannabis | | Sarpes Beverages |  |
| Harvest One Cannabis | | PhytoTech Therapeutics | |
| Harvest One Cannabis | | Delivra |  |
| Harvest One Cannabis | | LivCorp |  |
| Harvest One Cannabis | | LivVet |  |
| Harvest One Cannabis | | PortaPack |  |
| Heritage Cannabis | Umbral Energy |  |  |
| Heritage Cannabis |  | CannaCure |  |
| Heritage Cannabis |  | Purefarma Solutions | |
| Heritage Cannabis |  | CALYX Life Sciences | BriteLife Sciences |
| Heritage Cannabis |  | Voyage Cannabis | PhyeinMed |
| Heritage Cannabis |  | Mainstrain Market | |
| Heritage Cannabis |  | 333 Jarvis Realty |  |
| Heritage Cannabis |  | Heritage US Holdings | |
| Heritage Cannabis |  | Heritage (US) Cali |  |
| Heritage Cannabis |  | 5450 Realty |  |
| Heritage Cannabis |  | Heritage (US) Oregon | |
| Heritage Cannabis |  | Heritage (US) Colorado | |
| Heritage Cannabis |  | Opticann |  |
| Heritage Cannabis |  | Premium 5 |  |
| HEXO | The Hydropothecary | |  |
| HEXO |  | Keystone Isolation Technologies | |
| HEXO |  | Neal Up Brands |  |
| HEXO |  | Zenabis |  |
| HEXO |  | Newstrike Brands |  |
| Indiva | Thunder Sword Resources; Rainmaker Mining; Rainmaker Resources | | |
| Indiva |  | Vieva |  |
| Invictus MD Strategies | |  |  |
| Invictus MD Strategies | | Acreage Pharms |  |
| Invictus MD Strategies | | Future Harvest Development | |
| Invictus MD Strategies | | Leaf Wise |  |
| Invictus MD Strategies | | AB Laboratories |  |
| Invictus MD Strategies | | AB Ventures |  |
| Lotus Ventures | Strachan Resources | |  |
| MediPharm Labs | POCML 4 |  |  |
| MediPharm Labs |  | MPL |  |
| Mera Cannabis | Avana |  |  |
| Mera Cannabis |  | Garden Variety |  |
| Mera Cannabis |  | Ellevia |  |
| Mera Cannabis |  | CannaWay Clinic |  |
| MPX |  |  |  |
| MPX |  | Canveda |  |
| MPX |  | The CinG-X |  |
| MPX |  | Salus BioPharma |  |
| MPX |  | Biocannabis Products | |
| MPX |  | Spartan Wellness |  |
| MPX |  | MCLN |  |
| MPX |  | MPXI |  |
| MPX |  | HolyWorld | HolyWeed |
| MPX |  | Alphafarma Operations | |
| MPX |  | First Growth Holdings | |
| Namaste Technologies | Next Gen Metals |  |  |
| Namaste Technologies | | Australian Vaporizers | |
| Namaste Technologies | | CannMart |  |
| Namaste Technologies | | Findify |  |
| Namaste Technologies | | Namaste |  |
| Namaste Technologies | | NamasteMD |  |
| Northern Green |  |  |  |
| Organigram |  |  |  |
| Organigram |  | The Edibles & Infusions | The Edibles and Infusions |
| Sproutly |  |  |  |
| Sproutly |  | Toronto Herbal Remedies | |
| Sproutly |  | Infusion Biosciences | |
| Sproutly |  | SSM Partners |  |
| Sundial |  |  |  |
| Sundial |  | Sprout Technologies | |
| Sundial |  | KamCan Products |  |
| Sundial |  | Unipessoal |  |
| Sundial |  | NGBA-BC Holdings | |
| Tantalus Labs |  |  |  |
| Tantalus Labs |  | Tantalus Rx |  |
| TerrAscend |  |  |  |
| TerrAscend |  | The Apothecarium | |
| TerrAscend |  | Arise Bioscience |  |
| TerrAscend |  | Ilera Healthcare |  |
| TerrAscend |  | Valhalla Confections | |
| TerrAscend |  | State Flower |  |
| TerrAscend |  | HMS |  |
| TerrAscend |  | GuadCo |  |
| TerrAscend |  | KCR Holdings | Keystone Canna Remedies |
| THC Biomed |  |  |  |
| THC Biomed |  | THC BioMedical |  |
| THC Biomed |  | Clone Shipper |  |
| THC Biomed |  | THC2GO Dispensaries | |
| The Flowr |  |  |  |
| The Flowr |  | Holigen |  |
| The Flowr |  | RPK Biopharma |  |
| The Flowr |  | GreyCan |  |
| The Flowr |  | TCann |  |
| The Flowr |  | Terrace |  |
| The Flowr |  | Oransur |  |
| The Flowr |  | Terra Nova |  |
| The Flowr |  | Pharma Binoide |  |
| The Green Organic Dutchman | |  |  |
| The Green Organic Dutchman | | Medican Organic |  |
| The Green Organic Dutchman | | TGOD |  |
| The Green Organic Dutchman | | HemPoland |  |
| Valens |  |  |  |
| Valens |  | Straight Fire Consulting | |
| Valens |  | Southern Cliff Brands | Pommies Cider |
| Valens |  | LYF Food Technologies | |
| Valens |  | Green Roads |  |
| Tidal Health Solutions | |  |  |
| Tilray |  |  |  |
| Tilray |  | Natura Naturals |  |
| Tilray |  | Manitoba Harvest |  |
| Tilray |  | Dorada Ventures |  |
| Tilray |  | FHF Holdings |  |
| Tilray |  | High Park |  |
| Tilray |  | Pardal Holdings |  |
| Tilray |  | Fresh Hemp Foods |  |
| Tilray |  | National Cannabinoid Clinics | |
| Tilray |  | Privateer Evolution | |
| Village Farms |  |  |  |
| Village Farms |  | VF Clean Energy |  |
| Village Farms |  | Pure Sunfarms |  |
| VIVO Cannabis | Abcann |  |  |
| VIVO Cannabis |  | Canna Farms |  |
| VIVO Cannabis |  | Harvest Medicine |  |
| VIVO Cannabis |  | Green Earth Realty | |
| VIVO Cannabis |  | Patients' Choice Botanicals | |
| VIVO Cannabis |  | Universal Botanicals | |
| VIVO Cannabis |  | Beach Medical |  |
| VIVO Cannabis |  | Beacon Medical |  |
| Weed Me |  |  |  |
| Weed Me |  | CannaCurious |  |
| WeedMD |  |  |  |
| WeedMD |  | WMD Ventures |  |
| WeedMD |  | Starseed |  |
| WeedMD |  | North Star Wellness | |
