# Supplementary material for: Cannabis companies and the sponsorship of scientific research: A cross-sectional Canadian case study
Source: PLoS One. 2023 Jan 10;18(1):e0280110. doi: 10.1371/journal.pone.0280110 (PMC9831296; doi:10.1371/journal.pone.0280110)
Supplement: S2 Table — (DOCX) [file pone.0280110.s003.docx]

**S3 Table. List of PubMed searches**

| 1 | 333 Jarvis Realty[Conflict of Interest Statements] |
| --- | --- |
| 2 | 5450 Realty[Conflict of Interest Statements] |
| 3 | AB Laboratories[Conflict of Interest Statements] |
| 4 | AB Ventures[Conflict of Interest Statements] |
| 5 | Abba Medix[Conflict of Interest Statements] |
| 6 | Abcann[Conflict of Interest Statements] |
| 7 | ACB Captive Insurance[Conflict of Interest Statements] |
| 8 | Ace Valley[Conflict of Interest Statements] |
| 9 | Acreage Pharms[Conflict of Interest Statements] |
| 10 | AgMedica Bioscience[Conflict of Interest Statements] |
| 11 | AgriMed Botanicals[Conflict of Interest Statements] |
| 12 | Alberta Craft Cannabis[Conflict of Interest Statements] |
| 13 | Aleafia[Conflict of Interest Statements] |
| 14 | Algarithm Ingredients[Conflict of Interest Statements] |
| 15 | Alphafarma Operations[Conflict of Interest Statements] |
| 16 | Apollo Applied Research[Conflict of Interest Statements] |
| 17 | Aqualitas[Conflict of Interest Statements] |
| 18 | Arise Bioscience[Conflict of Interest Statements] |
| 19 | Atlas Biotechnologies[Conflict of Interest Statements] |
| 20 | Atlas Growers[Conflict of Interest Statements] |
| 21 | Atlas Thrive[Conflict of Interest Statements] |
| 22 | Aurora Cannabis[Conflict of Interest Statements] |
| 23 | Aurora Nordic Cannabis[Conflict of Interest Statements] |
| 24 | Australian Vaporizers[Conflict of Interest Statements] |
| 25 | AV Cannabis[Conflict of Interest Statements] |
| 26 | Avalite Sciences[Conflict of Interest Statements] |
| 27 | Avana[Conflict of Interest Statements] |
| 28 | BATAVIA BIO PROCESSING[Conflict of Interest Statements] |
| 29 | Beach Medical[Conflict of Interest Statements] |
| 30 | Beacon Medical[Conflict of Interest Statements] |
| 31 | Beckley Canopy Therapeutics[Conflict of Interest Statements] |
| 32 | BeeHigh Vital Elements[Conflict of Interest Statements] |
| 33 | BeeHighVE[Conflict of Interest Statements] |
| 34 | Benchmark Botanics[Conflict of Interest Statements] |
| 35 | BH![Conflict of Interest Statements] |
| 36 | Biocannabis Products[Conflict of Interest Statements] |
| 37 | BioSteel Sports Nutrition[Conflict of Interest Statements] |
| 38 | Black Birch Capital Acquisition[Conflict of Interest Statements] |
| 39 | Bower Therapies[Conflict of Interest Statements] |
| 40 | BriteLife Sciences[Conflict of Interest Statements] |
| 41 | C3 Cannabinoid Compound Company[Conflict of Interest Statements] |
| 42 | CALYX Life Sciences[Conflict of Interest Statements] |
| 43 | Canabo Medical[Conflict of Interest Statements] |
| 44 | Canada Bond Biotechnology[Conflict of Interest Statements] |
| 45 | Canada House Clinics[Conflict of Interest Statements] |
| 46 | Canada House Wellness Group[Conflict of Interest Statements] |
| 47 | Canamo y Fibras Naturales[Conflict of Interest Statements] |
| 48 | Canna Farms[Conflict of Interest Statements] |
| 49 | CannaCure[Conflict of Interest Statements] |
| 50 | CannaCurious[Conflict of Interest Statements] |
| 51 | CannaWay Clinic[Conflict of Interest Statements] |
| 52 | CannMart[Conflict of Interest Statements] |
| 53 | CannTrust[Conflict of Interest Statements] |
| 54 | CannTX Life Sciences[Conflict of Interest Statements] |
| 55 | Canopy Growth[Conflict of Interest Statements] |
| 56 | Canveda[Conflict of Interest Statements] |
| 57 | Capital Pool Company[Conflict of Interest Statements] |
| 58 | Clone Shipper[Conflict of Interest Statements] |
| 59 | Coldstream Manufacturing[Conflict of Interest Statements] |
| 60 | Coldstream Real Estate Holdings[Conflict of Interest Statements] |
| 61 | Cronos[Conflict of Interest Statements] |
| 62 | CTI Holdings[Conflict of Interest Statements] |
| 63 | Delivra[Conflict of Interest Statements] |
| 64 | Dorada Ventures[Conflict of Interest Statements] |
| 65 | Dream Products[Conflict of Interest Statements] |
| 66 | East Coast Tween[Conflict of Interest Statements] |
| 67 | EB TRANSACTION[Conflict of Interest Statements] |
| 68 | EB Transaction[Conflict of Interest Statements] |
| 69 | Ellevia[Conflict of Interest Statements] |
| 70 | Elmcliffe Investments[Conflict of Interest Statements] |
| 71 | Emblem[Conflict of Interest Statements] |
| 72 | Emerald Health[Conflict of Interest Statements] |
| 73 | Eve & Co[Conflict of Interest Statements] |
| 74 | Eve and Co[Conflict of Interest Statements] |
| 75 | FHF Holdings[Conflict of Interest Statements] |
| 76 | Findify[Conflict of Interest Statements] |
| 77 | First Growth Holdings[Conflict of Interest Statements] |
| 78 | Flying High Brands[Conflict of Interest Statements] |
| 79 | Fresh Hemp Foods[Conflict of Interest Statements] |
| 80 | Future Harvest Development[Conflict of Interest Statements] |
| 81 | Garden Variety[Conflict of Interest Statements] |
| 82 | GOOD BUDS[Conflict of Interest Statements] |
| 83 | Green Earth Realty[Conflict of Interest Statements] |
| 84 | Green Roads[Conflict of Interest Statements] |
| 85 | GreenTec[Conflict of Interest Statements] |
| 86 | Grey Bruce Farms[Conflict of Interest Statements] |
| 87 | GreyCan[Conflict of Interest Statements] |
| 88 | GrowWise Health[Conflict of Interest Statements] |
| 89 | GTEC Holdings[Conflict of Interest Statements] |
| 90 | GuadCo[Conflict of Interest Statements] |
| 91 | H2 Biopharma[Conflict of Interest Statements] |
| 92 | Harvest Medicine[Conflict of Interest Statements] |
| 93 | Harvest One Cannabis[Conflict of Interest Statements] |
| 94 | HemPoland[Conflict of Interest Statements] |
| 95 | Heritage (US) Cali[Conflict of Interest Statements] |
| 96 | Heritage (US) Colorado[Conflict of Interest Statements] |
| 97 | Heritage (US) Oregon[Conflict of Interest Statements] |
| 98 | Heritage Cannabis[Conflict of Interest Statements] |
| 99 | Heritage US Holdings[Conflict of Interest Statements] |
| 100 | HEXO[Conflict of Interest Statements] |
| 101 | High Park[Conflict of Interest Statements] |
| 102 | HIP Developments[Conflict of Interest Statements] |
| 103 | HIP NY Developments[Conflict of Interest Statements] |
| 104 | HMS[Conflict of Interest Statements] |
| 105 | Holigen[Conflict of Interest Statements] |
| 106 | HolyWeed[Conflict of Interest Statements] |
| 107 | HolyWorld[Conflict of Interest Statements] |
| 108 | Hortican[Conflict of Interest Statements] |
| 109 | Ilera Healthcare[Conflict of Interest Statements] |
| 110 | Indiva[Conflict of Interest Statements] |
| 111 | Infusion Biosciences[Conflict of Interest Statements] |
| 112 | Invictus MD Strategies[Conflict of Interest Statements] |
| 113 | IsoCanMed[Conflict of Interest Statements] |
| 114 | JuJu Joints[Conflict of Interest Statements] |
| 115 | KamCan Products[Conflict of Interest Statements] |
| 116 | KCR Holdings[Conflict of Interest Statements] |
| 117 | Keystone Canna Remedies[Conflict of Interest Statements] |
| 118 | Keystone Isolation Technologies[Conflict of Interest Statements] |
| 119 | Knalysis Technologies[Conflict of Interest Statements] |
| 120 | Lakessence[Conflict of Interest Statements] |
| 121 | Leaf Wise[Conflict of Interest Statements] |
| 122 | Les Serres Vert Cannabis[Conflict of Interest Statements] |
| 123 | LivCorp[Conflict of Interest Statements] |
| 124 | LivVet[Conflict of Interest Statements] |
| 125 | Lotus Ventures[Conflict of Interest Statements] |
| 126 | LW Capital Pool[Conflict of Interest Statements] |
| 127 | LYF Food Technologies[Conflict of Interest Statements] |
| 128 | Mainstrain Market[Conflict of Interest Statements] |
| 129 | Manitoba Harvest[Conflict of Interest Statements] |
| 130 | Marijuana For Trauma[Conflict of Interest Statements] |
| 131 | MCLN[Conflict of Interest Statements] |
| 132 | Medican Organic[Conflict of Interest Statements] |
| 133 | MediPharm Labs[Conflict of Interest Statements] |
| 134 | Mera Cannabis[Conflict of Interest Statements] |
| 135 | Milk Capital[Conflict of Interest Statements] |
| 136 | MPL[Conflict of Interest Statements] |
| 137 | MPX[Conflict of Interest Statements] |
| 138 | MPXI[Conflict of Interest Statements] |
| 139 | Namaste Technologies[Conflict of Interest Statements] |
| 140 | Namaste[Conflict of Interest Statements] |
| 141 | NamasteMD[Conflict of Interest Statements] |
| 142 | National Cannabinoid Clinics[Conflict of Interest Statements] |
| 143 | Natura Naturals[Conflict of Interest Statements] |
| 144 | Natural MedCo[Conflict of Interest Statements] |
| 145 | Neal Up Brands[Conflict of Interest Statements] |
| 146 | Newstrike Brands[Conflict of Interest Statements] |
| 147 | Next Gen Metals[Conflict of Interest Statements] |
| 148 | NGBA-BC Holdings[Conflict of Interest Statements] |
| 149 | North Star Wellness[Conflict of Interest Statements] |
| 150 | Northern Green[Conflict of Interest Statements] |
| 151 | Opticann[Conflict of Interest Statements] |
| 152 | Oransur[Conflict of Interest Statements] |
| 153 | Organigram[Conflict of Interest Statements] |
| 154 | Original BC[Conflict of Interest Statements] |
| 155 | Pardal Holdings[Conflict of Interest Statements] |
| 156 | Patients' Choice Botanicals[Conflict of Interest Statements] |
| 157 | Peace Naturals Project[Conflict of Interest Statements] |
| 158 | Peloton Pharmaceuticals[Conflict of Interest Statements] |
| 159 | Pharma Binoide[Conflict of Interest Statements] |
| 160 | PhyeinMed[Conflict of Interest Statements] |
| 161 | PhytoTech Therapeutics[Conflict of Interest Statements] |
| 162 | POCML 4[Conflict of Interest Statements] |
| 163 | Pommies Cider[Conflict of Interest Statements] |
| 164 | PortaPack[Conflict of Interest Statements] |
| 165 | POS[Conflict of Interest Statements] |
| 166 | Potanicals Green Growers[Conflict of Interest Statements] |
| 167 | Premium 5[Conflict of Interest Statements] |
| 168 | Privateer Evolution[Conflict of Interest Statements] |
| 169 | Pure Sunfarms[Conflict of Interest Statements] |
| 170 | Purefarma Solutions[Conflict of Interest Statements] |
| 171 | Rainmaker Mining[Conflict of Interest Statements] |
| 172 | Rainmaker Resources[Conflict of Interest Statements] |
| 173 | Redwood[Conflict of Interest Statements] |
| 174 | Reef Organic[Conflict of Interest Statements] |
| 175 | Reliva[Conflict of Interest Statements] |
| 176 | Royal City Cannabis[Conflict of Interest Statements] |
| 177 | RPK Biopharma[Conflict of Interest Statements] |
| 178 | Salus BioPharma[Conflict of Interest Statements] |
| 179 | Sarpes Beverages[Conflict of Interest Statements] |
| 180 | Satipharm[Conflict of Interest Statements] |
| 181 | Sindica Global Institute for Cannabis Research & Innovation[Conflict of Interest Statements] |
| 182 | Solidus Standard[Conflict of Interest Statements] |
| 183 | Southern Cliff Brands[Conflict of Interest Statements] |
| 184 | Spartan Wellness[Conflict of Interest Statements] |
| 185 | Spectre Labs[Conflict of Interest Statements] |
| 186 | Spectrum Biomedical[Conflict of Interest Statements] |
| 187 | Spectrum Labs[Conflict of Interest Statements] |
| 188 | Spectrum Therapeutics[Conflict of Interest Statements] |
| 189 | Sprout Technologies[Conflict of Interest Statements] |
| 190 | Sproutly[Conflict of Interest Statements] |
| 191 | SSM Partners[Conflict of Interest Statements] |
| 192 | Starseed[Conflict of Interest Statements] |
| 193 | State Flower[Conflict of Interest Statements] |
| 194 | Steadystem Solutions[Conflict of Interest Statements] |
| 195 | Storz & Bickel[Conflict of Interest Statements] |
| 196 | Storz and Bickel[Conflict of Interest Statements] |
| 197 | Strachan Resources[Conflict of Interest Statements] |
| 198 | Straight Fire Consulting[Conflict of Interest Statements] |
| 199 | Sundial[Conflict of Interest Statements] |
| 200 | Supreme Cannabis[Conflict of Interest Statements] |
| 201 | T-Bird Pharma[Conflict of Interest Statements] |
| 202 | Tantalus Labs[Conflict of Interest Statements] |
| 203 | Tantalus Rx[Conflict of Interest Statements] |
| 204 | TCann[Conflict of Interest Statements] |
| 205 | Terra Nova[Conflict of Interest Statements] |
| 206 | Terrace[Conflict of Interest Statements] |
| 207 | TerrAscend[Conflict of Interest Statements] |
| 208 | TGOD[Conflict of Interest Statements] |
| 209 | Thanos Holdings[Conflict of Interest Statements] |
| 210 | THC Biomed[Conflict of Interest Statements] |
| 211 | THC BioMedical[Conflict of Interest Statements] |
| 212 | THC Pharm GmbH Health Concept[Conflict of Interest Statements] |
| 213 | THC2GO Dispensaries[Conflict of Interest Statements] |
| 214 | The Apothecarium[Conflict of Interest Statements] |
| 215 | The CinG-X[Conflict of Interest Statements] |
| 216 | The Edibles & Infusions[Conflict of Interest Statements] |
| 217 | The Edibles and Infusions[Conflict of Interest Statements] |
| 218 | The Flowr[Conflict of Interest Statements] |
| 219 | The Green Organic Dutchman[Conflict of Interest Statements] |
| 220 | The Hydropothecary[Conflict of Interest Statements] |
| 221 | The Longevity Project[Conflict of Interest Statements] |
| 222 | The Supreme Cannabis Company[Conflict of Interest Statements] |
| 223 | This Works! Products[Conflict of Interest Statements] |
| 224 | Thunder Sword Resources[Conflict of Interest Statements] |
| 225 | Thunderbird Biomedical[Conflict of Interest Statements] |
| 226 | Tidal Health Solutions[Conflict of Interest Statements] |
| 227 | Tilray[Conflict of Interest Statements] |
| 228 | Toronto Herbal Remedies[Conflict of Interest Statements] |
| 229 | Tumbleweed Farms[Conflict of Interest Statements] |
| 230 | Tweed[Conflict of Interest Statements] |
| 231 | TWP[Conflict of Interest Statements] |
| 232 | Umbral Energy[Conflict of Interest Statements] |
| 233 | Unipessoal[Conflict of Interest Statements] |
| 234 | United Greeneries[Conflict of Interest Statements] |
| 235 | Universal Botanicals[Conflict of Interest Statements] |
| 236 | Valens[Conflict of Interest Statements] |
| 237 | Valhalla Confections[Conflict of Interest Statements] |
| 238 | Verdelite[Conflict of Interest Statements] |
| 239 | Verdélite[Conflict of Interest Statements] |
| 240 | VF Clean Energy[Conflict of Interest Statements] |
| 241 | Vieva[Conflict of Interest Statements] |
| 242 | Village Farms[Conflict of Interest Statements] |
| 243 | VIVO Cannabis[Conflict of Interest Statements] |
| 244 | Voyage Cannabis[Conflict of Interest Statements] |
| 245 | Wachstum Produce GP[Conflict of Interest Statements] |
| 246 | Weed Me[Conflict of Interest Statements] |
| 247 | WeedMD[Conflict of Interest Statements] |
| 248 | Whistler Medical Marijuana[Conflict of Interest Statements] |
| 249 | Wild Cove Smoke[Conflict of Interest Statements] |
| 250 | WMD Ventures[Conflict of Interest Statements] |
| 251 | Wyn Metals[Conflict of Interest Statements] |
| 252 | Zenabis[Conflict of Interest Statements] |
| 253 | Zenalytic Laboratories[Conflict of Interest Statements] |
| 254 | Zeus Cannabinoids[Conflict of Interest Statements] |
