# Supplementary material for: Cannabis companies and the sponsorship of scientific research: A cross-sectional Canadian case study
Source: PLoS One. 2023 Jan 10;18(1):e0280110. doi: 10.1371/journal.pone.0280110 (PMC9831296; doi:10.1371/journal.pone.0280110)
Supplement: S3 Table — (DOCX) [file pone.0280110.s004.docx]

**S4 Table. Coding manual**

| **Variable** | **Coding scheme/Description** | **Notes** |
| --- | --- | --- |
| Included | Y: Yes, included  N: none of the sampled cannabis companies are in COI statement  M: investigate company name further | *M may require further investigation into whether the company listed in the disclosure is in fact a cannabis company* |
| Companies | List company names | *List all for-profit cannabis companies mentioned in COI or funding statements or acknowledgments and separate by semi-colons* |
| Article_type | ER: Empirical research (Trials, Observational Studies, Research Letters etc.)  PR: Published study protocol  CR: Empirical, n of 1, case reports/studies  SR: Systematic review/Meta-analysis  CO: Commentary/Non-systematic reviews/Perspective/Opinions/Editorials  NA: Where entry was not included | *Systematic review is defined as a literature review with a reproducible search strategy (e.g. search terms, inclusion/exclusion criteria, flow diagram) and/or titles itself a “systematic review.” Search does not have to be exhaustive. Meta-analyses may or may not also have a systematic review. “Non-systematic” reviews do not have a methodology.* |
| COI_type_CAN | GR: Grant (related to the person and/or past work and not to the current work)  PE: Personal fees (consulting, advisory, speakers, honoraria, travel)  NF: Non-financial support (lab agreements, ongoing writing support etc.)  PA: Patents/copyrights/royalties  OW: Ownership or stocks  MISC: COIs disclosed that do not meet definition of COI or funding  None: No COI declared (per our definition)  No information: no COI statement  NS: declared company name but not COI type  NA: Where entry was not included | ***For Cannabis COIs:*** *According to ICMJE taxonomy and IOM definition of COI. Thus, all COI_types refer to relationships with* ***individual*** *authors that represent* ***secondary*** *interests that pose a* ***risk of bias*** *to the research. COI_type* ***does not*** *include employment or salary support for the current work; this should be entered under* ***funding_type.*** *Similarly, grant funding or non-financial support for the current work should be entered under* ***funding_type****.* |
| COI_type_OTH | GR: Grant (related to the person and/or past work and not to the current work)  PE: Personal fees (consulting, advisory, speakers, honoraria, travel)  NF: Non-financial support (lab agreements, ongoing writing support etc.)  PA: Patents/copyrights/royalties  OW: Ownership or stocks  MISC: COIs disclosed that do not meet definition of COI  None: Statement of none  No information: no COI statement  NS: declared company name but not COI type  NA: Where entry was not included | ***For other COIs:*** *According to ICMJE taxonomy and IOM definition of COI. Thus, all COI_types refer to relationships with* ***individual*** *authors that represent* ***secondary*** *interests that pose a* ***risk of bias*** *to the research. COI_type* ***does not*** *include employment or salary support for the current work; this should be entered under* ***funding_type.*** *Similarly, 0067rant funding or non-financial support for the current work should be entered under* ***funding_type****.* |
| COI_disclosure | Quoted verbatim  NA: Where entry was not included | *Information may be located under disclosures or under “acknowledgments” or other sections.* |
| Funding_source | GO: Government/not-for-profit  FP: For-profit  MIX: Mixed (for-profit plus not-for-profit)  None  No information  NA: Where entry was not included | *Any funding* ***for the current work****, including grants, employment, salary support, fellowships, statement of generic “research support” and non-financial support (e.g. providing product, or tests), should be classified, whether or not it is explicitly disclosed (e.g. industry-affiliated authors have for-profit funding_type).* |
| Funding_type_CAN | EM: Employment    GR: grant, fellowship/salary support, “research support”  NF: product or test, data, non-financial research materials only  None: Statement of None  No information (statement missing) | *Specific to the* ***cannabis*** *company* |
| Funding_type_OTH | EM: Employment    GR: grant, fellowship/salary support, “research support”  NF: product or test, data, non-financial research materials  None: Statement of none  No information (statement missing) | *Specific to* ***other*** *funding sources* |
| Funding_disclosure | Quoted verbatim  No information: Statement is missing  NA: Where entry was not included | *Information may be located under disclosures or under acknowledgments. Also include statements that describe the “role of the funder.”* |
| Authors_affiliation type | ACAD: academia, university, teaching hospital, hospital  GOV: Government  COM: For-profit company  NGO: Non-governmental, not-for-profit  MIX: Multiple affiliations  OTH: Other |  |
| Authors_location | Country name | *List authors’ country affiliation; separate multiple countries by semi-colons* |
| Is_Animal | Y: Yes  N: No  NA: Where entry was not included | *Yes includes whole animals, animal specimens, or cell cultures (i.e. not human subjects).* |
| Is_Plant | Y: Yes  N: human, animal study  NA: entry not included | *Yes includes whole plants, plants specimens, or cell cultures from plants (i.e. not human subjects).* |
| Is_Cannabis_Focus | Y: studies which focus on cannabis  N: all other articles  NA: entry was not included | *Code “yes” if authors discuss cannabis and it is mentioned by name (even generically) in the title or abstract.*  *Code “N” if cannabis is not mentioned in the title or abstract.* |
| Is_Interventional | Y: empirical, prospective intervention studies including any trials or experiments with cannabis  N: all other empirical research  NA: entry was not included, not empirical, a case report or a plant study | *Code if the article_type =* ***empirical research.*** *“Yes” includes any empirical study where the investigators* ***prospectively*** *introduced an* ***intervention*** *(humans or animals)* |
| Is_Formulation | DR: Dried cannabis (smoked, inhalation, vaped)  ED: Edible (e.g., brownie, lollipop, cookie, chocolate)  TOP: Topical (e.g. Lotion, spray, oil, or cream)  CAP: Capsule  SPRAY: oromucosal spray  MULTP: multiple formulations  OT: Other formulation  NS: Formulation not mentioned  NA: entry not included or plant or animal study or article not cannabis focused |  |
| Topic_focus | Specify the topic focus | *Copy and paste the study’s topic focus* |
| Pop_Descript | Description of sample’s social location  NA: for animal or plant studies | *Copy and paste most relevant text describing the population of focus. This could be the population from which the sample was drawn in terms of social location (race/ethnicity, SES, sexual orientation, geographic location, special population (e.g. veterans) etc) and key* ***inclusion/exclusion criteria*** *(if available) OR population focus of commentary or systematic review.* |
| Study_location | [Country name]  NA: systematic reviews, commentaries, non-empirical papers, animal or plant studies | *Country where data were collected; only for empirical articles with human subjects* |
| Sample_size | Report n  NA: for non-empirical, plant or animal studies | *Number of included participants* |
| Report_age | Y: Age of sample reported  N: Age of sample not reported  NA: if not included, not empirical, plant or animal study |  |
| Sample_age |  | *State age range and any relevant stats (mean, media, SD)* |
| Report_gender | Y: gender of sample reported  N: gender of sample not reported  NA: if not included, not empirical, plant or animal study |  |
| Sample_gender |  | *State % (e.g. male % vs female, men, % women, % non-binary (as reported))* |
| Report_Race | Y: Race/ethnicity of sample reported  N: Race/ethnicity not reported  NA: if not included, not empirical, plant or animal study |  |
| Sample_race |  | *Extract relevant stats* |
| Report_sexual orientation | Y: Sample sexual orientation reported  N: Sample sexual orientation not reported  NA: if not included, not empirical, plant or animal study |  |
| Sample_sexual_orientation |  | *Extract relevant stats* |
| Report_SES | Y: Sample socioeconomic status reported  N: Sample socioeconomic status not reported  NA: if not included, not empirical, plant or animal study | *Include things like employment status, housing status, income* |
| Sample_SES |  | *Extract relevant stats* |
| Report_education | Y: Sample education level reported  N: Sample education level not reported  NA: if not included, not empirical, plant or animal study |  |
| Sample_education |  | *Extract relevant stats* |
