# Supplementary material for: Cannabis companies and the sponsorship of scientific research: A cross-sectional Canadian case study
Source: PLoS One. 2023 Jan 10;18(1):e0280110. doi: 10.1371/journal.pone.0280110 (PMC9831296; doi:10.1371/journal.pone.0280110)
Supplement: Attachment — Submitted filename: PLoS ONE R1 Response to reviewers.docx [file pone.0280110.s006.docx]

Quinn Grundy

Assistant Professor

Suite 130, 155 College St, Toronto, ON, M5T1P8

[quinn.grundy@utoronto.ca](mailto:quinn.grundy@utoronto.ca)

+1-647-913-7075

*PLoS ONE*

Re: Submission of revised manuscript entitled, "Canadian cannabis companies and the sponsorship of scientific research: A cross-sectional Canadian case study"

Dear Dr. Baogui Xin,

Thank you for the opportunity to revise the above-named manuscript and we thank you for the encouraging and constructive reviews.

At this revision stage, we confirm that the authors have declared that no competing interests exist.

We have also addressed the issue of data availability and have included our full dataset now as a Supplementary File, which we cite within the manuscript. Our revised data availability statement should read:

“The full dataset underlying this analysis is available in Supplementary File 4.”

Thank you for your time in consideration of this submission.

Sincerely,

Quinn Grundy,

on behalf of

Daphne Imahori

Shreya Mahajan

Gord Garner

Roberta Timothy

Abhimanyu Sud

Sophie Soklaridis

Daniel Z Buchman

| **Comments** | **Response** | **Changes made** |
| --- | --- | --- |
| **Reviewer 1** | | |
| Reviewer #1: The methods are rigorous and best-practice for meta-research. The paper is well-written and results are not overstated. | Thank you for this encouraging review. | No change. |
| My only recommendations is that I think the data availability statement is inadequate for PLOS One. Data should be deposited in a public repository. Given this is meta-research, I don't see any barrier to this (i.e. no patient information, IRB oversight, etc.) | We have now made our full, coded dataset available in conformance with the PLOS One data availability policy. We now include the full dataset as a Supplementary Excel File accompanying the manuscript. | Please see Supplementary File 4. |
| **Reviewer 2** | | |
| Reviewer #2: “Canadian cannabis companies and the sponsorship of scientific 1 research: A cross-sectional Canadian case study” offers a systematic study of the study sponsorship and conflicts of interest supported by the Canadian cannabis sector. The study deploys a novel search protocol focusing on identified Canadian cannabis firms and PubMed-indexed conflicts of interest statements. The study collected 156 articles with a named cannabis company in the COI statement conducted three data collection exercises. The first collected key data on all articles and focused on study type (research, commentary, review), author location, COI disclosures, study subjects (human, plants, non-human animals) and the presence or absence of specific cannabis content in the study. A subsequent content analysis of the 72 cannabis-focused articles identifies article topics including conditions addressed (where appropriate) and other article foci (e.g., harm reduction, cannabis harms, preclinical studies, etc). The final analysis extracts participant demographics data for the 87 collected human studies. This is one of the first systematic studies of COI in research on medical applications of cannabis. The study design is appropriate and rigorous. The submitted results provide an important foundation for future research in this area. I recommend publication following a few revisions. | Thank you for this encouraging and constructive review. We have addressed each of your comments in the rows below. | No change. |
| The content analysis would be improved if combined with the COI analysis. Specifically, given the overall focus of the article, I want to know more about which article topics were associated with cannabis sponsorship or COIs. This information could be added to Table 2 and discussed in the results section. | In analyzing our data, we initially also thought to analyze the main topic of sampled articles by presence of author COI or cannabis company study support. However, due to the diversity of topics and missing funding declarations (in 10% of cannabis-focused articles), we found that this sub-analysis resulted in such small sample sizes (often an n of 1) in each cell that the analysis lost its meaning. Because our inclusion criteria specified that all articles had to disclose *some* cannabis company relationship and we did not include a comparison group (e.g. cannabis-focused articles that had no cannabis company ties), we feel that a sub-analysis may be misleading or fail to paint a full picture of patterns in the cannabis industry research agenda. However, we appreciate this comment and felt that it may be more helpful to provide further detail on the *type* of cannabis industry sponsorship or nature of authors’ relationships with cannabis companies. For example, we note that research grants are not the most prevalent form of study support – rather, cannabis company employees are conducting research and/or are members of author teams in 35% of these articles, which may also account for authors’ declarations of cannabis company stock or ownership interests (in 21% of articles). We now include this information alongside Table 2. | We have added the following descriptive analysis just preceding Table 2:  “Among articles with a cannabis focus, 25 (35%) included cannabis company employee authors; 17 (24%) received study support from a cannabis company in the form of a research grant; and 12 (17%) received non-financial support in the form of product, devices, data, technical, or writing support. Authors of the majority of cannabis-focused articles (56/72, 78%) declared a conflict of interest with a cannabis company; prevalent declarations included receipt of personal fees from cannabis companies (47/72, 65%), cannabis company research sponsorship for past projects (16/72, 22%), and ownership interest or stocks in cannabis companies, including the sponsor (15/72, 21%). |
| As replicability with R can be a special challenge, I would recommend describing the R version and key packages (and versions) in a bit more detail. | We have added additional detail on the R version and key packages as requested. We used R and the package easyPubMed to extract article metadata (as indexed by PubMed). We now include the R version and cite the specific easyPubMed package in the Methods section. We also provide further detail and citations for each of the packages used for data cleaning (rlist, tidyr, stringr, and dplyr). | We have added the following detail to the Methods section under “Data extraction and analysis”:  “Using the R version 4.1.0 and the package easyPubMed [version 2.13], we automatically extracted article metadata, as indexed by PubMed, which included: author names, author affiliations, title, abstract, journal, article type, DOI, and verbatim conflict of interest statement. We also used the packages rlist, tidyr, stringr, and dplyr for data cleaning.” |
| The limitations should be more specific about the gaps in coverage for COI reporting on PubMed where open access journals are over-represented. Highlighting this limitation is important to showcase the likelihood that there are even greater cannabis COI rates than currently observable in the current study. | Thank you for this point. In the revised limitations section, we have noted that our study likely underestimates the extent of Canadian cannabis company research sponsorship or financial relationships with researchers. Specifically, we also note that previous research analyzing the conflict of interest statements of articles published between 2016 and 2018 and indexed in PubMed found that only one of the highest h-index medical journals, for example, reported conflict of interest information to PubMed (i.e. the *BMJ* which is also open access). Thus, this descriptive analysis necessarily provides a partial perspective of the phenomenon of Canadian cannabis company research activities. | Please see the revised text in the Discussion sub-section “Strengths and limitations”:  “Further, it is unknown the extent to which PubMed meta-data related to conflict of interest is complete or comprehensive in terms of indexed journals; thus, our study likely also underestimates the extent of Canadian cannabis company research sponsorship or financial relationships with researchers. For example, previous research analyzing the conflict of interest statements of articles published between 2016 and 2018 and indexed in PubMed found that only one of the highest h-index medical journals reported conflict of interest information to PubMed.[28] Thus, this descriptive analysis necessarily provides a partial perspective of the phenomenon of Canadian cannabis company research activities.” |
| Inter-rater reliability scores using a chance-correcting statistics, reported alongside percent agreement, would provide readers with a more comprehensive understanding of the agreement picture. | In this study, we adopted and then adapted a coding manual from a previous study of the prevalence of disclosed conflict of interest in biomedical research articles (see Grundy et al., JAMA, 2018). In replicating the previous studies methods, we elected not to calculate inter-rater reliability statistically, as the key variables of interest (e.g. type of conflict of interest, nature of study support) required a high degree of interpretation and codes were not necessarily mutually exclusive (e.g. an author team may disclose multiple types of COI).  Therefore, we used percent agreement as one measure of coder reliability to assess the clarity of our revised coding manual during a pilot phase. In coding the full sample, we decided to have two team members, working independently, code all data, with a third author available to resolve disagreements. These methods are more consistent with systematic review and other meta-research methods that involve systematic data extraction and interpretive coding procedures. This process also ensured that coding was comprehensive, resolving discrepancies or omissions through discussion and a consensus-process. | We have clarified our coding decisions in the Methods section:  “We piloted the coding manual on a random 10% sample of included articles, with 85% agreement. We elected to code the remaining articles in pairs of authors working independently to ensure comprehensiveness and accuracy, with discrepancies reviewed by a third author and final coding reached by consensus.” |
| All data should be made available in the supplementary materials or in a data repository per PLoS One guidelines. | We have now made our full, coded dataset available in conformance with the PLOS One data availability policy. We now include the full dataset as a Supplementary Excel File accompanying the manuscript. | Please see Supplementary File 4. |
